# A Multifunctional Au/CeO_2_-Mg(OH)_2_ Catalyst for One-Pot Aerobic Oxidative Esterification of Aldehydes with Alcohols to Alkyl Esters

**DOI:** 10.3390/nano11061536

**Published:** 2021-06-10

**Authors:** Seulgi Lim, Seungdon Kwon, Nagyeong Kim, Kyungsu Na

**Affiliations:** Department of Chemistry, Chonnam National University, Gwangju 61186, Korea; swing9sg@gmail.com (S.L.); kwon950515@gmail.com (S.K.); brightest24@gmail.com (N.K.)

**Keywords:** multifunctional catalyst, oxidative esterification, aldehyde, alkyl ester, Au catalyst, strong metal–support interaction

## Abstract

Au nanoparticles bound to crystalline CeO_2_ nanograins that were dispersed on the nanoplate-like Mg(OH)_2_, denoted as Au/CeO_2_-Mg(OH)_2_, were developed as the highly active and selective multifunctional heterogeneous catalyst for direct oxidative esterification of aldehydes with alcohols to produce alkyl esters under base-free aerobic conditions using oxygen or air as the green oxidants. Au/CeO_2_-Mg(OH)_2_ converted 93.3% of methacrylaldehyde (MACR) to methyl methacrylate (MMA, monomer of poly(methyl methacrylate)) with 98.2% selectivity within 1 h, and was repeatedly used over eight recycle runs without regeneration. The catalyst was extensively applied to other aldehydes and alcohols to produce desirable alkyl esters. Comprehensive characterization analyses revealed that the strong metal–support interaction (SMSI) among the three catalytic components (Au, CeO_2_, and Mg(OH)_2_), and the proximity and strong contact between Au/CeO_2_ and the Mg(OH)_2_ surface were prominent factors that accelerated the reaction toward a desirable oxidative esterification pathway. During the reaction, MACR was adsorbed on the surface of CeO_2_-Mg(OH)_2_, upon which methanol was simultaneously activated for esterifying the adsorbed MACR. Hemiacetal-form intermediate species were subsequently produced and oxidized to MMA on the surface of the electron-rich Au nanoparticles bound to partially reduced CeO_2−x_ with electron-donating properties. The present study provides new insights into the design of SMSI-induced supported-metal-nanoparticles for the development of novel, multifunctional, and heterogeneous catalysts.

## 1. Introduction

Oxidation and esterification are important organic synthetic processes in the industrial production of various fine and bulk chemicals [1,2,3]. Various oxygenated substrates can be oxidized or esterified to obtain desirable compounds using homogeneous or heterogeneous catalysts [4,5,6,7]. A typical heterogeneous catalyst can be fabricated by supporting active catalytic components on supporting materials that generally have large surface areas. Various nanocrystalline metallic species are used as the primary catalytic components and are supported on nanoporous metal oxides. The Au nanoparticle-based nanostructured catalyst is a representative oxidation catalyst that often exhibits higher activity and better selectivity to a desirable product under milder reaction conditions than those of other noble metal catalysts such as Pt-group metals (PGMs) [8,9,10,11]. Similarly, various nanostructured acidic or basic metal-oxide-catalysts can be used for the esterification of aldehydes with alcohols to synthesize ester compounds that possess industrial versatility as a useful commodity chemical [12,13,14].

**Scheme 1:**
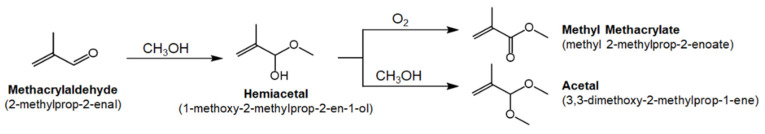
Direct oxidative esterification of methacrylaldehyde (MACR) with methanol under aerobic conditions using oxygen as the oxidant. MACR can be esterified with methanol to produce hemiacetal species as the intermediate that can be competitively either oxidized to methyl methacrylate (MMA) as the desirable alkyl ester product or over-esterified with methanol to acetal species as undesirable by-products.

In several chemical reactions, heterogeneous catalysts control the reaction process by modifying the activation energy and molecular adsorption geometry, which ultimately control the reaction rate and product selectivity. Therefore, the development of innovative catalysts with improved catalytic performances is a crucial research goal in the field of catalysis [15,16]. The design of multifunctional catalysts that reduce the complexity of multi-step reaction processes is also a top priority [17,18]. One such achievement involves the oxidative esterification of methyl methacrylaldehyde (MACR) with methanol to produce methyl methacrylate (MMA), which is an important monomer of poly(methyl methacrylate) (PMMA) [19,20,21,22,23]. Prior to this invention, the industrial production of MMA was carried out via the commercialized acetone cyanohydrin (ACH) process that employed toxic hydrocyanic acid and generated a large amount of waste chemicals such as ammonium bisulfate and acidic wastewater [21,22,23,24]. Therefore, the direct oxidative esterification of MACR to MMA under aerobic conditions is highly desirable (Scheme 1) compared to the ACH process in terms of atom economy, energy efficiency, green chemistry, and sustainability [12,24,25].

Various precious metals (Pt, Pd, and Au) supported on metal oxides have been investigated in recent decades as heterogeneous catalysts for increasing reaction efficiencies [9,26,27,28]. Among the various metal nanoparticle catalysts, the Au nanoparticle catalyst is considered relatively more active and selective than PGM-based catalysts for this reaction [10,11]. Bimetallic structures with Au-based heterogeneous catalysts and secondary metal components have been prepared and investigated to improve the catalytic activity and product selectivity [6,29,30]. The effects of metal oxides as catalyst supports and the modification of catalyst morphology have also been studied to achieve similar results [7,31]. The catalytic behavior of Au metal nanoparticles on the supported catalyst structure is critically governed by the metal–support interactions at the interface between Au and the metal oxide supports [22,32,33]. The activity, product selectivity, and catalyst stability can be influenced by the sizes, shapes, and chemical states of the Au metal nanoparticles, and are synergistically connected to the properties of the metal oxide supports [33,34,35,36]. Various nanostructured metal oxide materials such as TiO_2_, Al_2_O_3_, CeO_2_, SiO_2_, Ga_2_O_3_, La_2_O_3_, hydrotalcite, and polymers have been investigated as supports for improving catalytic performances [13,37,38,39,40,41,42,43,44,45,46].

The key issues regarding research on Au-based catalysts involve increasing the activity and product selectivity [28,47,48], removing the liquid base additive from the reaction solution [7,40,49], designing mild reaction conditions that require low energy consumption [31,42], and enhancing the lifetime by improving the catalyst stability [28,48]. Considering these issues, a rationally designed, active, selective, and stable heterogeneous Au-based catalyst supported on CeO_2_-Mg(OH)_2_ was fabricated in the present study to facilitate the one-pot oxidative esterification of aldehydes with alcohols for producing alkyl esters under aerobic conditions in the absence of base additives. This catalyst, Au/CeO_2_-Mg(OH)_2_, achieved an MACR conversion of 93.3% and an MMA selectivity of 98.2% with sustainable catalyst reuse and without significant deactivation of up to eight repeated non-regenerative runs. Comprehensive reaction analyses with characterization of the catalyst properties, compared to those of Au nanoparticles supported exclusively either on CeO_2_ or on Mg(OH)_2_ revealed that the resultant catalyst structure featured Au nanoparticles that were strongly bound to the crystalline CeO_2_ nanograins that were dispersed on the surface of the Mg(OH)_2_ nanoplate; this interaction was found to be critical in achieving a good catalytic performance.

## 2. Materials and Methods

### 2.1. Material Preparation

The supported Au catalysts were synthesized via the deposition–precipitation (DP) or co-precipitation (CP) methods using the procedures described below. All the chemicals were used as obtained without further purification.

Au nanoparticles were pre-synthesized with an organic capping agent to initiate the process of synthesizing Au nanoparticles supported on Mg(OH)_2_. In a typical synthesis of Au nanoparticles, 0.30 g of HAuCl_4_·3H_2_O (Alfa Aesar) and 0.15 g poly(vinyl alcohol) (PVA; Daejung Chemicals & Metals, Busan, Korea, MW = 1500) were stirred in 300.0 mL of distilled water in an ice bath for 30 min. Subsequently, 50.0 mL of an aqueous solution containing 0.144 g of NaBH_4_ (Kanto Chemical, Tokyo, Japan) was added to the previous solution under vigorous magnetic stirring for 1 h. Subsequently, 4.85 g of MgO (Sigma Aldrich, St. Louis, MO, USA) was added to the aqueous solution containing the pre-synthesized PVA-capped Au nanoparticles. After the suspension was vigorously stirred in an ice bath for 2 h, the resulting solid sample was filtered, washed with distilled water, and dried overnight at 60 °C. The MgO phase was almost completely hydrated to Mg(OH)_2_ during the synthesis of the supported Au catalysts, as confirmed by x-ray diffraction (XRD).

To synthesize Au nanoparticles supported on CeO_2_, 0.30 g of HAuCl_4_·3H_2_O, and 9.19 g of Ce(NO_3_)_3_·6H_2_O (Daejung Chemicals & Metals, Busan, Korea) were mixed in 85.0 mL of distilled water containing 4.43 g of Na_2_CO_3_ (Daejung Chemicals & Metals, Busan, Korea) to maintain a pH of 10. The aqueous solution was stirred at 25 °C for 1 h, and the resulting solid sample was filtered, washed with hot water, and dried overnight at 100 °C. The dried powder was subsequently calcined at 400 °C for 4 h under air flow.

To synthesize Au nanoparticles supported on CeO_2_-Mg(OH)_2_, 55.44 g of Ce(NO_3_)_3_·6H_2_O and 55.62 g of Mg(NO_3_)_2_·6H_2_O (Daejung Chemicals & Metals, Busan, Korea) were dissolved together in 100.0 mL of distilled water containing 72.5 g of citric acid monohydrate (Daejung Chemicals & Metals, Busan, Korea). The aqueous solution was stirred at 80 °C for 5 h, and the solution was subsequently placed in a Petri dish in an oven at 100 °C overnight. The resulting fluffy polymeric mixture in the Petri dish was collected, ground to a fine powder, and calcined at 450 °C for 9 h under air flow to yield CeO_2_-Mg(OH)_2_, upon which the Au nanoparticles were supported via the DP method. An aqueous solution of HAuCl_4_ (2.55 × 10^−3^ M) was slowly added to the solution containing CeO_2_-Mg(OH)_2_ under vigorous stirring. The resulting mixture was stirred at 65 °C for 3 h, and the solid sample was subsequently filtered and washed with distilled water to remove the leftover chloride anions. The resulting sample was dried overnight at 60 °C.

The obtained Au nanoparticles supported on Mg(OH)_2_, CeO_2_, and CeO_2_-Mg(OH)_2_ were denoted as 3AuM, 3AuC, and 3AuCM, respectively, where the loading of Au nanoparticles was fixed at 3 wt.%. The representative catalyst loading of 3 wt.% was selected as a result of optimization of the catalytic performance parameters with various Au loadings such as 1, 2, 3, 4, and 5 wt.%. It should be noted that the actual loadings of the Au nanoparticles in these samples were 2.80, 2.63, and 2.36 wt.%, respectively, as determined by ICP-OES (Table 1).

### 2.2. Material Characterization

XRD patterns of the synthesized catalysts were obtained using a Rigaku MiniFlex 600 apparatus with Cu Kα radiation (λ = 0.1541 nm) at 40 kV and 15 mA (600 W). All measurements were performed under ambient conditions with a step size of 0.02°, scanning rate of 4° min^−1^, and 2 θ range of 15–70°. The content of Au in the catalysts was determined via inductively coupled plasma optical emission spectroscopy (ICP-OES; Avio 500, PerkinElmer, Waltham, MA, USA). Nitrogen adsorption/desorption isotherms were measured using a Micromeritics ASAP 2020 volumetric analyzer at 77 K. All investigated catalysts were degassed at 300 °C for 3 h prior to the measurements. The surface areas were derived using the Brunauer–Emmett–Teller (BET) theory, and the total pore volumes and pore size distributions were obtained from the adsorption branches using the Barrett–Joyner–Halenda (BJH) algorithm [50,51]. Transmission electron microscopy (TEM) images were obtained using a Tecnai F-20 (Philips, Cambridge, MA, USA) instrument operating at 200 kV (lattice resolution; 0.19 nm). X-ray photoelectron spectroscopy (XPS) was performed using a K-ALPHA^+^ (Thermo Fisher Scientific, Waltham, MA, USA) setup equipped with a monochromatic Al Kα source connected to a 128-channel detector. Prior to the measurements, all investigated catalysts were reduced under H_2_/Ar flows of 60 and 50 mL min^−1^, respectively, at 350 °C for 2 h using a ramping rate of 3 °C min^−1^. The reduced samples in powder form were placed on a stainless steel sample holder and fixed with a carbon tape, and were subsequently analyzed under a vacuum of 5 × 10^−9^ mbar (pass energy of 200 eV for survey spectra and 50 eV for narrow spectra, take-off angle of 60°). The spectra were fitted using Gaussian–Lorentzian curves after performing the Shirley baseline correction.

H_2_ temperature-programmed reduction (TPR) measurements were carried out using a BEL-CAT (BEL, Osaka, Japan) analyzer. All investigated samples were degassed using a stream of Ar for 1 h at 300 °C in a quartz cell, followed by cooling to 30 °C. The samples were treated with 5% H_2_/Ar flows with increasing cell temperatures from 50 to 850 °C (ramping rate of 5 °C min^−1^). H_2_ consumption was measured using a thermal conductivity detector (TCD). Temperature-programmed desorption–mass spectrometry of ammonia and carbon dioxide (NH_3_- and CO_2_-TPD–MS) was performed using the BEL-CAT analyzer. To facilitate this, the sample was first heated to 300 °C under He flow, held for 1 h, and subsequently cooled to 50 °C. A 5% NH_3_/He mixture or CO_2_ flow was introduced for 1 h. Subsequently, the sample was flushed under He flow to eliminate weakly physisorbed NH_3_ (or CO_2_). The NH_3_–TPD–MS (or CO_2_-TPD–MS) profile was obtained under He flow from 100 to 650 °C.

The dispersion of Au on the catalyst was estimated via the amount of CO chemisorbed using the BEL-CAT analyzer and a mixture of 5% CO/He. The sample was heated to 300 °C in H_2_ and subsequently held for 1 h. Thermogravimetric analysis (TGA) was performed using a PerkinElmer STA 6000 (PerkinElmer, Waltham, MA, USA) simultaneous thermal analyzer.

### 2.3. Catalytic Reaction

The catalytic reaction was performed using a Teflon-lined stainless-steel autoclave containing the reactant solution dissolved in an organic solvent. To initiate the reaction, the Au-supported catalyst was reduced under H_2_ and Ar flow (20 and 20 mL min^−1^, respectively) at 300 °C for 5 h with a ramping rate of 2.5 °C min^−1^. In a typical reaction process, 6 mL (0.0725 mol) of MACR (TCI, >90%) was dissolved in a solution containing 14.668 mL of methyl alcohol (Daejung Chemicals & Metals, Busan, Korea) (MACR:MeOH = 1:5) and 1.5 g of the catalyst. Prior to the catalytic test, the reactor was purged with O_2_ and subsequently pressurized with O_2_ or air to the designated pressure (6–12 bar); the temperature of the reactor was subsequently increased to the designated temperature. The reaction was performed at a stirring speed of 600 rpm. After the catalytic reaction, the reactor was cooled to below room temperature in an ice bath, and the reaction solution was separated from the solid catalyst using a syringe filter. The collected solution was analyzed using a gas chromatograph (GC, Younglin YL6500, Anyang, Korea) equipped with a flame ionization detector (FID) with a capillary column (DB-5, length: 30 m; diameter: 0.32 mm; thickness: 1.5 μm) in the presence of ethanol as an internal standard. The reaction products were also confirmed via a gas chromatography–mass spectrometry setup (GC–MS, Agilent 7890A, Wilmington, DE, USA) with a mass selective detector (5975B). For the recyclability tests, the spent solid catalyst was washed with methanol and hexane using a centrifuge (10,000 rpm for 10 min, twice).

## 3. Results and Discussion

### 3.1. Materials Characterization

**Figure 1:**
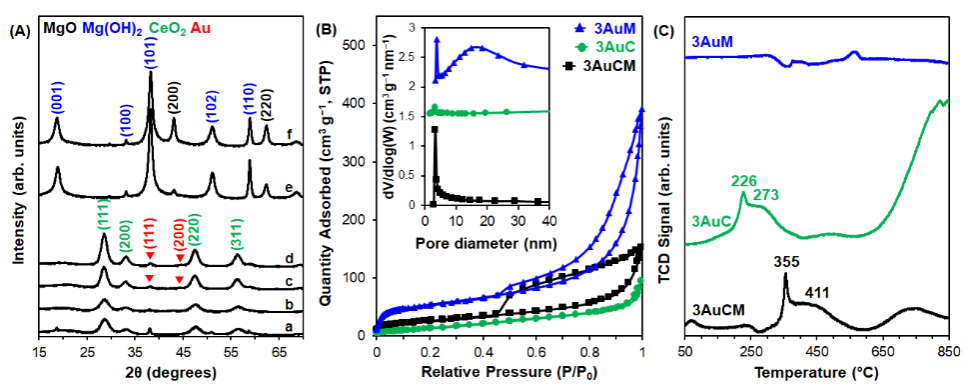
(**A**) X-ray diffraction patterns, (**B**) N_2_ adsorption/desorption isotherms with pore size distributions (inset), and (**C**) H_2_ temperature-programmed reduction profiles of the 3AuM, 3AuC, and 3AuCM catalysts. The x-ray diffraction patterns of (a, c, e) and (b, d, f) correspond to the samples before and after H_2_ treatment, respectively.

The XRD patterns of 3AuCM in Figure 1A show broad peaks of (111), (200), (220), and (311) corresponding to CeO_2_, which is the most substantial component of 3AuCM (63.9 wt.% of CeO_2_ in AuCM); less intense peaks of (001), (101), (102), and (110) corresponding to Mg(OH)_2_ (33.8 wt.% Mg(OH)_2_ in AuCM) were also observed. The contents of CeO_2_ and Mg(OH)_2_ in the 3AuCM catalyst were determined as 63.9 and 33.8 wt.%, respectively, by ICP-OES. Rietveld analysis of the 3AuCM sample revealed that the compositions of CeO_2_ and Mg(OH)_2_ were 72% and 28%, respectively. The thermal treatment at 300 °C in an H_2_ environment induced a partial dehydration of Mg(OH)_2_ to MgO, as evidenced by the increase in XRD reflections at 2 θ values of 43° and 62°, which correspond to the (200) and (220) domains of MgO [52]; however, no appreciable changes were observed in the crystalline phases of CeO_2_. In addition, characteristic reflections corresponding to the crystalline phase of metallic Au nanoparticles were observed after the H_2_ treatment, indicating the formation of nanocrystalline Au particles with adequate dispersion on the supporting material. In the case of 3AuC and 3AuM, typical intense XRD reflections corresponding to CeO_2_ and Mg(OH)_2_ were observed.

The porosity of the catalysts was analyzed using N_2_ adsorption isotherms (Figure 1B and Table 1). The results reveal that 3AuM possessed the largest mesoporosity, whereas 3AuC had the smallest porosity. The 3AuCM catalyst possessed a medium mesoporosity between those of 3AuM and 3AuC. The BET surface area and pore volume decreased in the following order: 3AuM > 3AuCM > 3AuC (Table 1). The effect of metal oxide supports on the reducibility of supported Au catalysts was also analyzed via H_2_-TPR (Figure 1C). Mg(OH)_2_ is known to be an almost non-reducible metal oxide [14,53], which can explain the fact that noteworthy signals representing H_2_ consumption were not observed in the 3AuM sample. In contrast, CeO_2_ is known to be a highly reducible metal oxide [14,54], and therefore the 3AuC sample exhibited a peak at a low reduction temperature of ~226–273 °C [55]. Interestingly, the shape of the H_2_-TPR profile of 3AuCM was similar to that of 3AuC, indicating that the reducible natures of 3AuCM and 3AuC are comparable. However, the reduction temperature shifted to a higher temperature of ~355–411 °C. This indicates that the presence of Mg(OH)_2_ in the 3AuCM catalyst decreases the reducibility, which can be attributed to the strong interaction between Mg(OH)_2_ and CeO_2_ in the ensembled structure [56].

**Figure 2:**
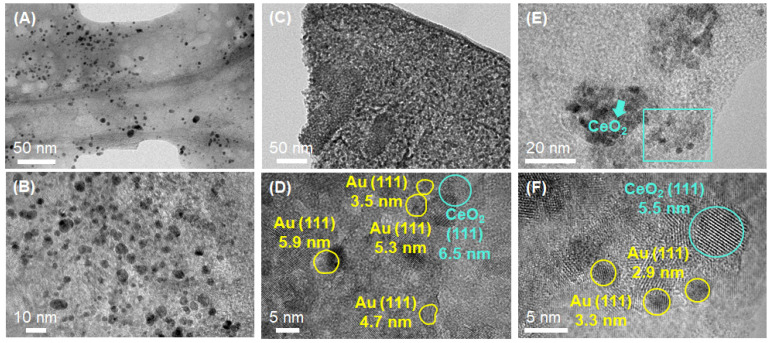
Transmission electron microscopy images of the (**A**,**B**) 3AuM, (**C**,**D**) 3AuC, and (**E**,**F**) 3AuCM catalysts at low- and high-magnification (top and bottom rows, respectively).

The TEM images in Figure 2 show the internal structures of the fabricated catalysts. For all three investigated Au catalysts, the Au nanoparticles were supported with a decent degree of dispersion (Table 1). The actual loading of Au nanoparticles in the three catalysts was in the 2.36–2.80 wt.% range, as determined via ICP, and their average sizes determined via TEM were in the 3.8–5.0 nm range. The average size of the Au nanoparticles increased in the following order: 3AuM (3.8 nm) < 3AuCM (4.1 nm) < 3AuC (5.0 nm); this trend was inversely proportional to that of the surface area of the supporting materials. A comparison of the TEM images of 3AuCM and 3AuM leads to important observations. At a similar Au loading, Au nanoparticles were uniformly dispersed on the nanoplate-like Mg(OH)_2_ surfaces. Due to the significant difference in the electron densities of Au and Mg, the Au nanoparticles in the 3AuM sample were clearly noticeable as dark black particles (Figure 2A,B). However, Au nanoparticles were not individually observed in the low-resolution images of the 3AuC and 3AuCM samples due to an overlap with the CeO_2_ phase (Figure 2C,E). However, the high-resolution TEM images revealed that the narrow (111) lattice planes of the Au nanoparticles epitaxially interacted with the wide (111) lattice planes of CeO_2_ (Figure 2D,F). Most of the Au nanoparticles were in proximity with CeO_2_ nanograins that possessed a domain size of 5–10 nm. This observation indicates that the Au nanoparticles preferably interacted with CeO_2_ more than with Mg(OH)_2_ due to the strong metal–support interaction (SMSI) at the interface between Au and CeO_2_.

**Figure 3:**
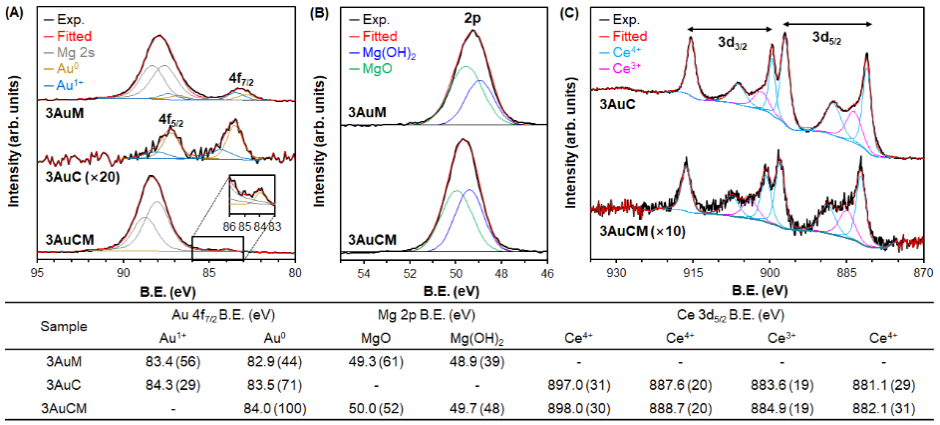
X-ray photoelectron profiles corresponding to the representative binding energy regions of (**A**) Mg 2s, Au^0^, and Au^1+^, (**B**) Mg(OH)_2_ and MgO, and (**C**) Ce^4+^ and Ce^3+^ for the 3AuM, 3AuC, and 3AuCM catalysts. The table below summarizes the binding energy values for the representative peaks, and the numbers in parentheses indicate the percentages of elements with different electronic states. The experimental and fitted XPS profiles are drawn in black and red colors, respectively.

The SMSI effect between Au and CeO_2_ can be evidenced via XPS, which also provides deeper insights into the electronic structures of the catalyst surfaces (Figure 3). The Au 4f_7/2_ peaks are generally observed in a binding energy range of 83–85 eV [41,57,58]. For the 3AuCM sample, a 4f_7/2_ peak appeared at 84.0 eV, which corresponded to the metallic Au(0) species (Figure 3A). A similar peak corresponding to metallic Au(0) was observed at 83.5 eV in the 3AuC sample. In the 3AuM sample, this peak was observed at a weaker binding energy of 82.9 eV. The stronger binding energy of Au in the 3AuCM and 3AuC samples can be attributed to the SMSI effect between Au and the CeO_2_ nanograins, whereas the weaker binding energy of Au in the 3AuM sample indicated weaker interactions between Au and the Mg(OH)_2_ surface [58]. In the Mg 2p region (Figure 3B), two peaks corresponding to MgO and Mg(OH)_2_ were observed in the 3AuM and 3AuCM samples. For the 3AuCM sample, the Mg 2p peaks appeared at 50.0 and 49.7 eV, and corresponded to MgO and Mg(OH)_2_, respectively; these peaks were present at weaker binding energies of 49.3 and 48.9 eV in the 3AuM sample. These results suggest that the Mg species in the 3AuCM sample strongly interacts with the CeO_2_ surface. The strong interaction between Mg and the Ce species in 3AuCM was also confirmed in a similar manner. Considering the Ce 3d region (Figure 3C), the Ce 3d_5/2_ peak of the reduced Ce species (Ce^3+^) appeared at 884.9 eV in 3AuCM and 883.6 eV in 3AuC. XPS analysis revealed that the combination of Au, CeO_2_, and Mg(OH)_2_ can induce strong interactions between each other at the interface, and therefore the 3AuCM sample possessed the highest binding energies for all the elements compared to those of the 3AuC and 3AuM samples. As confirmed via H_2_-TPR analysis (Figure 1C), 3AuCM exhibited an H_2_-TPR profile in a higher temperature region than that of 3AuC. This can be attributed to the strong binding of CeO_2_ nanograins to the non-reducible Mg(OH)_2_ surface, which is in line with the results from the XPS analysis.

### 3.2. Reaction Studies

The 3AuCM catalyst with strong interactions between its three catalytic components exhibited a notable catalytic behavior for the oxidative esterification of MACR with methanol under various reaction conditions (Table 2). NH_3_- and CO_2_-TPD–MS were used to confirm that all the supporting materials employed in this study (CeO_2_-Mg(OH)_2_, CeO_2_, Mg(OH)_2_) possessed surface acidity and/or basicity (Figure S1 in the Supplementary Materials and Table 1). Therefore, the acidic or basic sites on the catalyst surface can activate the esterification pathway as the first reaction step involving the conversion of MACR to hemiacetal in the absence of Au nanoparticles as the oxidation catalysts (entries 1–3 in Table 2) [59]. However, the bare support catalysts converted MACR with an MMA product selectivity of less than 3%. This result clearly suggests that the oxidation catalyst is indispensable for shifting the over-esterification pathway to the oxidative esterification route. As expected, 3AuCM exhibited a significantly high MACR conversion of 82.7% and a selectivity of 97.6% MMA, which corresponded to a turnover number (TON) and site-time yield (STY) of 1170 and 1142, respectively (entry 4 in Table 2). It should be noted that the H_2_ environment-based pre-treatment is necessary for facilitating close interactions between the catalytic components to enhance the catalytic activity. The non-reduced 3AuCM catalyst exhibited less activity with an MACR conversion of 52.1% (entry 5 in Table 2). Under the optimized reaction condition featuring a larger amount of the 3AuCM catalyst, 93.3% of MACR was converted with an MMA selectivity of 98.2% within 1 h (entry 6 in Table 2). MACR was fully converted within 1 h to yield 99.8% MMA when the 4AuCM catalyst was used under reaction conditions similar to those shown in entry 6 (not shown in Table 2). However, in terms of reaction efficiency, a loading of 3 wt.% of the Au nanoparticles on the CeO_2_-Mg(OH)_2_ surface was nearly optimal, compared to that with 4 wt.%. The final structure of MMA was also confirmed via ^1^H NMR (Figure S2 in the Supplementary Materials). The 3AuCM catalyst was also sufficiently active for the reaction under aerobic conditions instead of a pure O_2_ environment. Although a lesser amount of MACR was converted under aerobic conditions due to the lesser amount of oxidant in the air, the MMA selectivity remained at a high level of ~90% (entry 7 in Table 2). The effects of temperature and pressure on the catalytic performances of the 3AuCM catalyst were also investigated (Figure S3 in the Supplementary Materials). As the reaction temperature increased, the MACR conversion gradually increased; however, optimal performance was observed at 80 °C. Regardless of the reaction temperatures, MMA selectivity remained at nearly 100%. The optimal reaction result with respect to the O_2_ pressure was obtained at 9 bar.

The 3AuC and 3AuM catalysts also exhibited similar catalytic behaviors and converted MACR to yield MMA as the major product (entries 8 and 10 in Table 2). As the amounts of both catalysts in the reaction system increased, the MACR conversion increased and the MMA selectivity remained constant (entries 9 and 11 in Table 2). However, compared to the 3AuCM catalyst, both 3AuC and 3AuM exhibited slightly lower activities. In addition, GC analysis revealed that the carbon balances of 3AuC and 3AuM were 25% and 71%, respectively, whereas that of 3AuCM was 88% under similar reaction conditions (entries 4, 8, and 10 in Table 2). The unknown peaks observed at long retention times in the GC chromatogram for all catalysts possibly correspond to polymerized MMA, which has a larger molecular weight (Figure S4 in the Supplementary Materials). As a result, the carbon balances for the reactions with the 3AuC and 3AuM catalysts were smaller than that of the 3AuCM catalyst, suggesting that 3AuCM is a more active and selective catalyst.

The effect of Au loading on the catalyst surface was investigated using the Mg(OH)_2_ support, which possessed the largest mesoporosity (Table 2); this was done to exclude the possible effect of the formation of Au nanoparticles with different sizes on the Mg(OH)_2_ surface corresponding to changes in the Au loading. The reaction results revealed that the catalytic activity as well as MMA selectivity were critically influenced as the Au loading decreased from 3 to 2 and 1 wt.% on the Mg(OH)_2_ support (entries 11–13 in Table 2). When Au loading was reduced to 2 and 1 wt.%, stepwise decreases in both MACR conversion and MMA selectivity were observed (entries 12–13 in Table 2). When the 1AuM catalyst was employed, the MACR conversion and the MMA selectivity decreased to 31.7% and 84.3%, respectively (entry 13 in Table 2). This simple investigation on the effect of Au loading in the catalyst demonstrates that the balance of active sites between the oxidation and esterification sites is crucial for achieving a high MACR conversion with high MMA selectivity. The 4AuM catalyst, which supported 4 wt.% of the Au nanoparticles, was also investigated (not shown here); however, no significant changes were observed compared to the results with 3AuM, indicating that 3 wt.% was close to the maximum efficiency loading.

The 3AuCM catalyst exhibited excellent stability and decent recyclability, and also showcased sustainable catalytic performances during eight repetitive runs. The MACR conversion remained at ~90% during the eight repetitive reactions, whereas the MMA selectivity was maintained at ~98% (Figure S5 in the Supplementary Materials). No appreciable Au leaching was observed. It should be noted that no regeneration of the catalyst was carried out between the individual repetitive runs, which indicated that the 3AuCM catalyst possessed good efficiency and recyclability for the oxidative esterification of MACR with methanol to produce MMA. For comparative purposes, 3AuM was also tested for eight repetitive reactions; its activity notably decreased as the reaction was repeated (Figure S6 in the Supplementary Materials). TEM analysis revealed that the Au nanoparticles aggregated to form a large particle or leached out from the Mg(OH)_2_ surface, which can be attributed to the weak metal–support interaction between Au and Mg(OH)_2_ [60] (Figure S7 in the Supplementary Materials). In contrast, the 3AuCM catalyst maintained the initial size of the Au nanoparticles without significant aggregations; characteristic peaks corresponding to the large Au nanoparticles did not appear in the XRD pattern of the spent catalyst (Figures S8 and S9A in the Supplementary Materials). In addition, TGA and N_2_ adsorption analyses confirmed that no significant changes in the catalyst texture were observed (Figure S9B,C in the Supplementary Materials). The decent level of recyclability with framework stability can be attributed to the strong interactions of the three catalytic components (Au, CeO_2_, and Mg(OH)_2_) in 3AuCM.

The 3AuCM catalyst was subsequently employed as a general catalyst for the oxidative esterification of various aldehydes and alcohols to produce desirable ester products (Table 3). With MACR as the aldehyde reactant, the 3AuCM catalyst activated not only methanol, but also other alcohols such as n-butanol, 1-propanol, and iso-propanol. Depending on the alcohol reactant, the conversion of MACR varied; however, a desirable ester product was dominantly produced with more than 80% selectivity in all the reactions (entries 1–4 in Table 3). This level of broad alcohol scope for MACR conversion is practically significant because the resulting alkyl methacrylate products can be used as monomers or additives for the synthesis of poly(alkyl methacrylate) with tailored polymeric properties [61]. In addition to MACR, larger aldehydes such as benzaldehyde can be oxidatively esterified with methanol using the 3AuCM catalyst, and high conversion of benzaldehyde (59.4%) was achieved with 100% selectivity to a desirable ester product within 1 h (entry 5 in Table 3). Although the conversion of aldehydes reacting with larger alcohols was lower than the case of oxidative esterification between MACR and methanol, the conversion may possibly be enhanced by the increase in catalyst added in the reaction system or by the increase of reaction time. The extensive substrate applicability of the 3AuCM catalyst is a great advantage for practical applications and can be generalized further to react with various aldehydes and alcohols.

## 4. Conclusions

Three different types of supported Au nanoparticle catalysts with different degrees of SMSI were synthesized. The fabricated catalysts exhibited significantly different catalytic performances. XPS and H_2_-TPR analyses confirmed that the Au/CeO_2_-Mg(OH)_2_ catalyst showed the highest degree of SMSI compared to those of Au/CeO_2_ and Au/Mg(OH)_2_. The Au nanoparticles in the Au/CeO_2_-Mg(OH)_2_ catalyst were strongly bound to the surface of CeO_2_ nanograins that were dispersed on the Mg(OH)_2_ nanoplate via strong interactions. The combination of three different catalytic components in a single catalyst structure facilitated catalytic synergy for the oxidative esterification of aldehydes with alcohols to produce value-added alkyl esters. The SMSI at the interfaces of the three catalytic components and their close proximities accelerated the esterification of aldehydes with alcohols to produce a hemiacetal-form intermediate species in the first reaction step; the electron-rich Au nanoparticles bound to partially reduced CeO_2−x_ that possessed electron-donating properties, oxidizing the hemiacetal to a desirable alkyl ester in the second step. Consequently, the Au/CeO_2_-Mg(OH)_2_ catalyst exhibited a remarkably high and sustainable catalytic activity and product selectivity without losing its initial performance when compared to those of Au/CeO_2_ and Au/Mg(OH)_2_. The catalytic performances of SMSI-induced supported Au nanoparticle catalysts can provide scientific insights for the efficient design of highly active, selective, and stable multifunctional heterogeneous catalysts.

## Figures and Tables

**Table 1:** Physicochemical properties of the 3AuM, 3AuC, and 3AuCM catalysts.

	Au Content (wt. %) ^(a)^	Au Size (nm) ^(b)^	Au Dispersion (%) ^(c)^	S_BET_ (m^2^ g^−1^) ^(d)^	D_p_ (nm) ^(e)^	V_tot_ (cm^3^ g^−1^) ^(f)^	Amount of Acidic Sites (mmol g^−1^) ^(g)^			Amount of Basic Sites (mmol g^−1^) ^(h)^			
							A_T_	A_W_	A_S_	B_T_	B_w_	B_M_	B_S_
3AuM	2.80	3.8	30.8	187	3.8	0.58	0.401	0	0.401	0.028	0.007	0.014	0.007
3AuC	2.63	5.0	23.9	60	3.4	0.13	0	0	0	0.018	0.006	0.003	0.009
3AuCM	2.36	4.1	28.5	97	3.3	0.23	0.154	0.009	0.145	0.030	0.013	0.010	0.007

^(a)^ determined by ICP-OES; ^(b)^ determined by TEM image; ^(c)^ determined by mean size of Au; ^(d)^ S_BET_, BET surface area determined by the Brunauer–Emmet–Teller (BET) method; ^(e)^ D_p_, average pore diameter determined by the Barrett–Joyner–Halenda (BJH) method; ^(f)^ V_tot_, total pore volume;^(g)^ determined by NH_3_-TPD: A_T_, A_W_, and A_S_ are total, weak, and strong acid sites, respectively.; ^(h)^ determined by CO_2_-TPD: B_T_, B_W_, B_M_, and B_S_ are total, weak, medium, and strong basic sites, respectively.

**Table 2:** Reaction results of the aerobic oxidative esterification of MACR with methanol ^(a^^)^.

Entry	Catalyst	Weight (g)	M/R ^(b)^ Ratio (%)	MACR Conv. (%)	MMA Sel. (%)	Hemiacetal Sel. (%)	Acetal Sel. (%)	Y_MMA_ (%) ^(c)^	Carbon Balance (%) ^(d)^	TON ^(e)^	STY ^(f)^
1	MgO	1.5	-	30.9	0.0	40.2	59.8	0	22	-	-
2	CeO_2_	1.5	-	61.1	1.7	81.2	17.2	1.0	38	-	-
3	CeO_2_-MgO	1.5	-	33.8	2.6	54.1	43.4	0.9	10	-	-
4	3AuCM	1.5	0.315	82.7	97.6	1.6	0.8	80.7	88	1170	1142
5 ^(g)^	3AuCM	1.5	0.315	52.1	96.8	2.7	0.5	50.4	88	737	713
6	3AuCM	3.0	0.630	93.3	98.2	1.8	0.0	91.6	81	660	648
7 ^(^^h)^	3AuCM	1.5	0.315	40.0	89.9	4.5	5.5	36.0	80	566	510
8	3AuC	1.5	0.315	77.7	82.5	7.4	10.1	64.1	25	1203	993
9	3AuC	3.0	0.630	86.3	80.1	7.2	12.7	69.1	20	668	535
10	3AuM	1.5	0.315	66.5	94.3	2.3	3.4	62.7	71	735	693
11	3AuM	3.0	0.630	83.7	96.7	2.1	1.2	80.9	82	463	447
12	2AuM	1.5	0.210	62.5	87.3	2.6	10.1	54.5	75	1433	1250
13	1AuM	1.5	0.105	31.7	84.3	7.7	7.9	26.7	64	1382	1164

^(a^^)^ general reaction condition: methanol/MACR = 5:1, MACR 0.0725 mol, O_2_ pressure of 9 bar at 353 K for 1 h; ^(b^^)^ M/R ratio is metal(Au) to reactant ratio; ^(c^^)^ Y_MMA_, yield of MMA calculated by (MACR conv. × MMA sel./100); ^(d^^)^ carbon balance (total carbon content as a percentage) determined by GC; ^(e^^)^ TON (mol_MACR_ mol_Au_^−1^), turnover number calculated by (moles of MACR reacted/moles of active Au); ^(f^^)^ STY (mol_MMA_ mol_Au_^−1^ h^−1^), site-time yield calculated by (moles of MMA produced/moles of active Au/time); ^(g^^)^ Reaction result in entry 5 was obtained by using the 3AuCM catalyst before H_2_ treatment; ^(h^^)^ Reaction in entry 7 was carried out under air condition at 9 bar.

**Table 3:** Reaction results of the aerobic oxidative esterification of various aldehydes with different alcohols using the 3AuCM catalyst at 80 °C for 1 h under an O_2_ pressure of 9 bar. The conversion and selectivity with respect to a desirable alkyl ester were analyzed using a gas chromatograph equipped with a mass spectrometer.

Entry	Aldehyde	Alcohol	Product	Conversion/Selectivity (%) ^(a)^
1	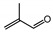	methanol	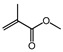	82.7/97.6
2 ^(b)^	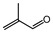	n-butanol	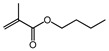	42.9/96.4
3 ^(c)^	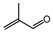	1-propanol	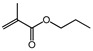	31.4/87.5
4 ^(d)^	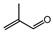	isopropyl alcohol	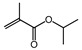	22.3/80.7
5 ^(e)^	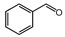	methanol	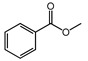	59.4/100

^(a)^ Conversion of aldehyde and ester selectivity were determined by GC–MS; ^(b)^ Entry 2: Butyl methacrylate (*m*/*z* = 69 (100), 87 (90), 41 (90), 56 (81)); ^(c)^ Entry 3: Propyl methacrylate (m/z = 43 (100), 31 (75), 55 (28), 69 (28), 83 (17), 127 (M^+^, 15)); ^(d)^ Entry 4: Isopropyl methacrylate (m/z = 69 (100), 45 (74), 87 (56), 58 (24), 31 (16)); ^(e)^ Entry 5: Methyl benzoate (*m*/*z* = 105 (100), 77 (57), 136 (38), 51 (20)).

## Supplementary Materials

The following are available online at https://www.mdpi.com/article/10.3390/nano11061536/s1, Figure S1: NH_3_ and CO_2_ TPD analysis of catalysts, Figure S2: ^1^H NMR data of reaction solution using 3AuCM, Figure S3: Oxidative esterification reaction results of 3AuCM, Figure S4: GC chromatograms of catalysts, Figure S5: Recycle tests of 3AuCM, Figure S6: Recycle tests of 3AuM, Figure S7: TEM images of 3AuM, Figure S8: TEM images of 3AuCM, Figure S9: XRD, TGA, and BET data of 3AuCM.
