# Supplementary material for: A Multifunctional Au/CeO2-Mg(OH)2 Catalyst for One-Pot Aerobic Oxidative Esterification of Aldehydes with Alcohols to Alkyl Esters
Source: Nanomaterials (Basel). 2021 Jun 10;11(6):1536. doi: 10.3390/nano11061536 (PMC8230364; doi:10.3390/nano11061536)
Supplement: Supplementary file 1 [file nanomaterials-11-01536-s001.zip › nanomaterials-1253513-supplementary.pdf]

Supplementary Material

A Multifunctional Au/CeO_2-$Mg(OH)_2$ Catalyst for One-Pot Aerobic Oxidative Esterification of Aldehydes with Alcohols to Alkyl Esters

Seulgi Lim †, Seungdon Kwon †, Nagyeong Kim and Kyungsu Na *

Department of Chemistry, Chonnam National University, Gwangju 61186, Korea; swing9sg@gmail.com (S.L.); kwon950515@gmail.com (S.K.); brightest24@gmail.com (N.K.)

* Correspondence: kyungsu_na@chonnam.ac.kr

† These authors contributed equally to this work.

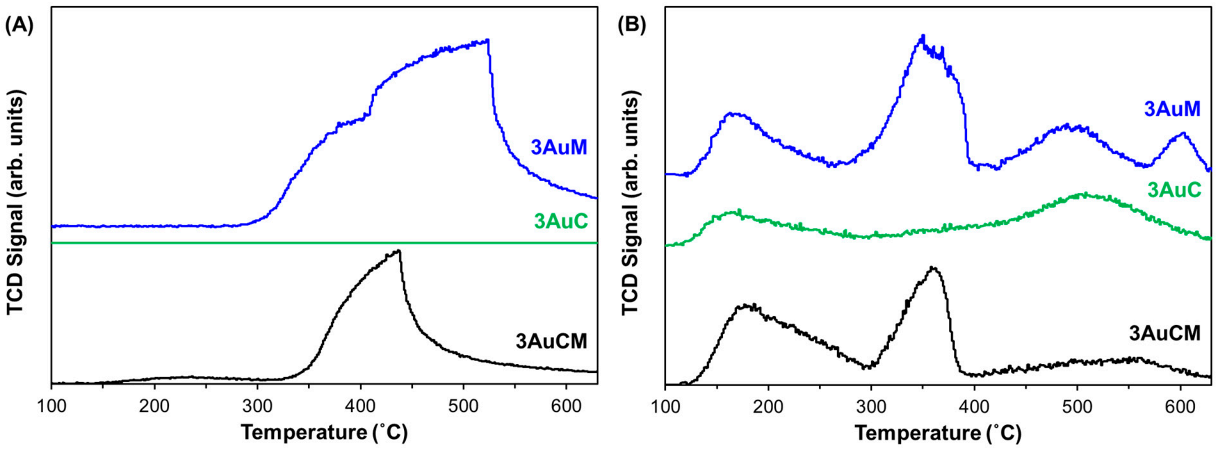

Figure S1. (**A**) NH_3-TPD and (**B**) CO_2-TPD profiles of 3AuM (blue), 3AuC (green), and 3AuCM (black).

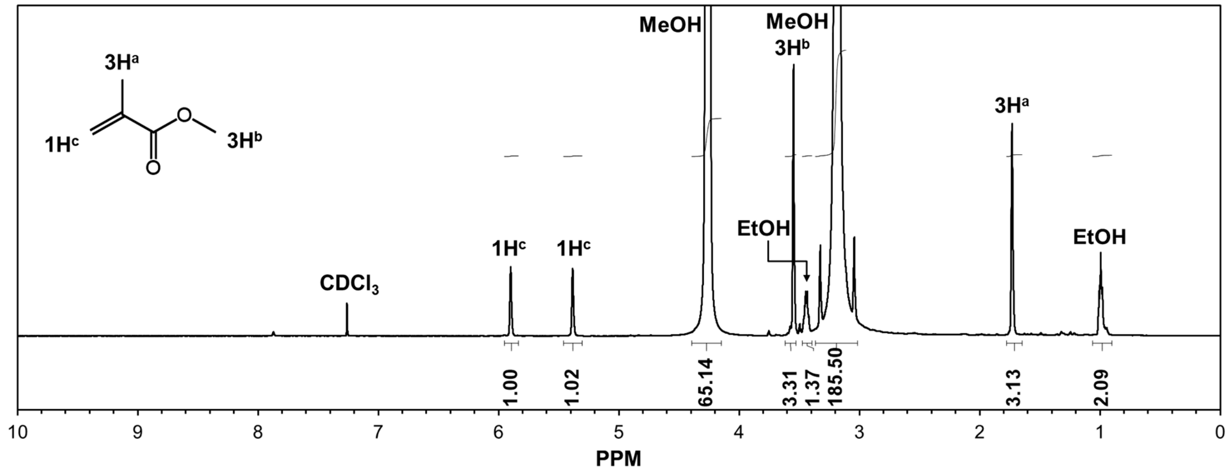

Figure S2. ^{1}H NMR results of the reaction solution described in Entry 6 in Table 2, which indicates a highly pure MMA product. $CDCl_3$ is employed as the deuterated solvent and ethanol is added in the reaction solution as the internal standard.

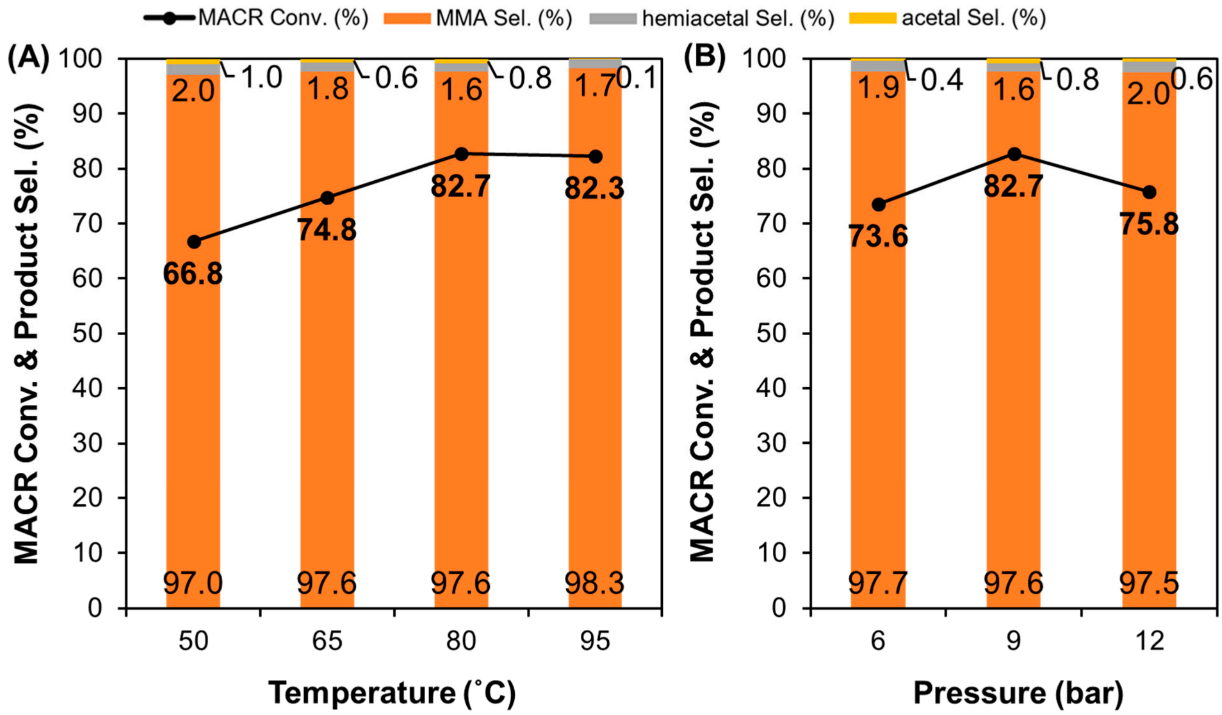

Figure S3. Effect of (**A**) reaction temperatures and (**B**) pressures with respect to the 3AuCM catalyst on the oxidative esterification of methacrylaldehyde with methanol. The reaction is performed at various reaction temperatures of 50, 65, 80, and 95 °C, and O_2 pressures of 6, 9, and 12 bar.

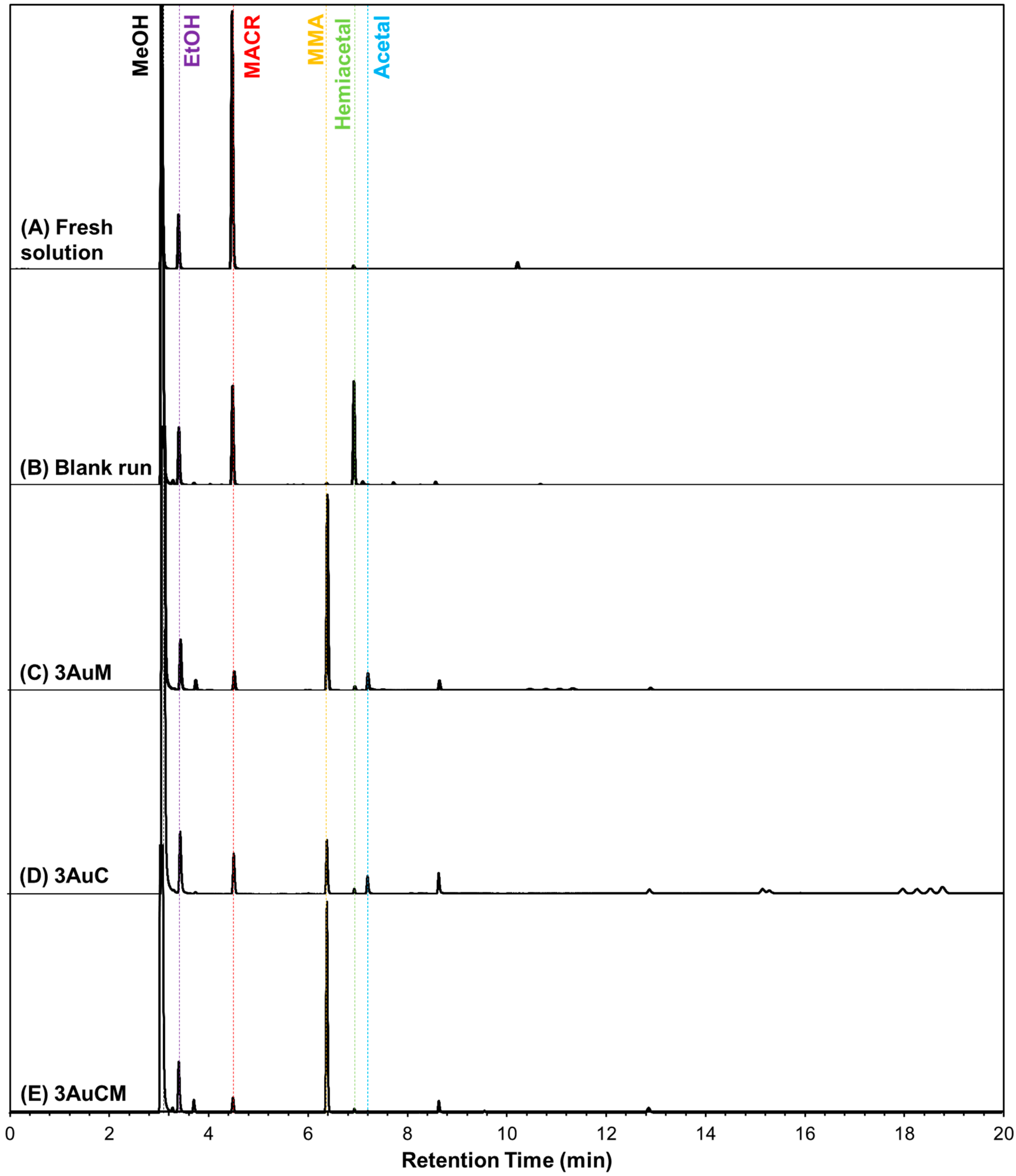

Figure S4. GC chromatograms of (**A**) a fresh reaction solution before the reaction in the absence of the catalyst, reaction solutions at 80 °C for 1 h (**B**) in the absence of the catalyst or in the presence of (**C**) 3AuM, (**D**) 3AuC, and (**E**) 3AuCM.

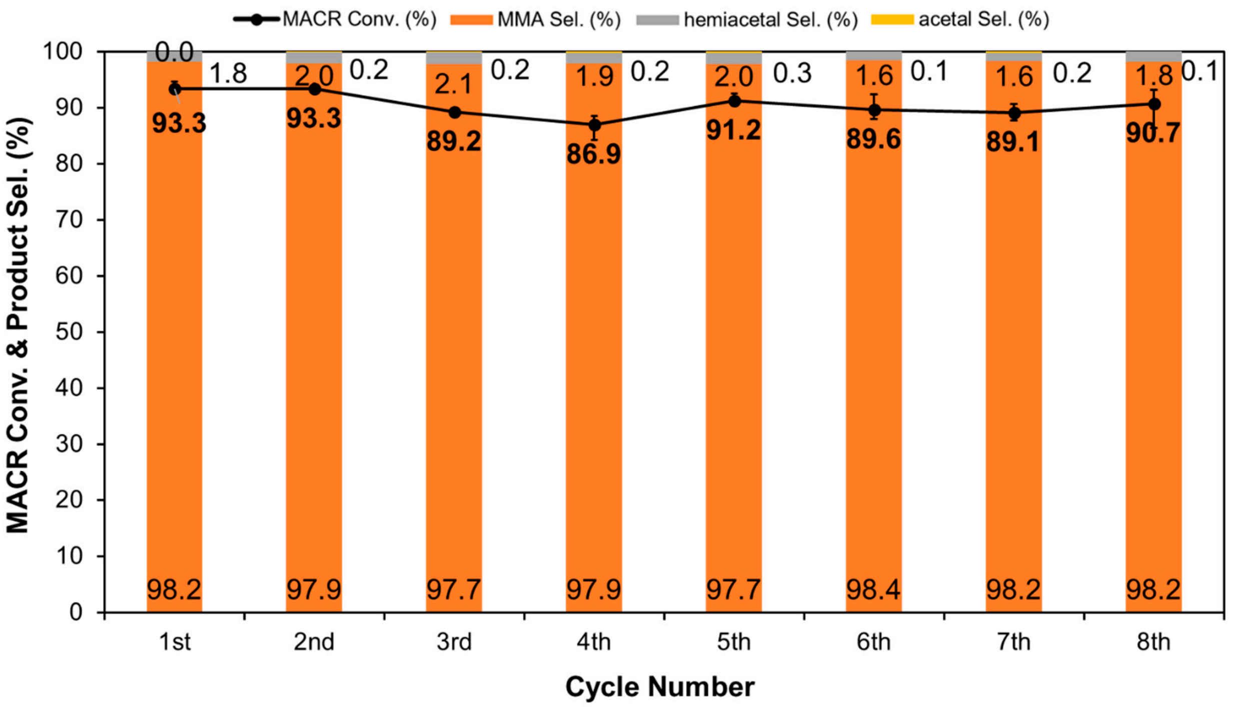

Figure S5. Recycle tests of 3AuCM during the oxidative esterification of methacrylaldehyde with methanol for eight repetitive runs. Black dots indicate the conversion of methacrylaldehyde with error bars derived from three measurements. Orange bars with numbers at the bottom indicate the selectivity of methyl methacrylate. Each reaction is carried out at 80 °C for 1 h and follows reaction conditions similar to those in Entry 6 in Table 2.

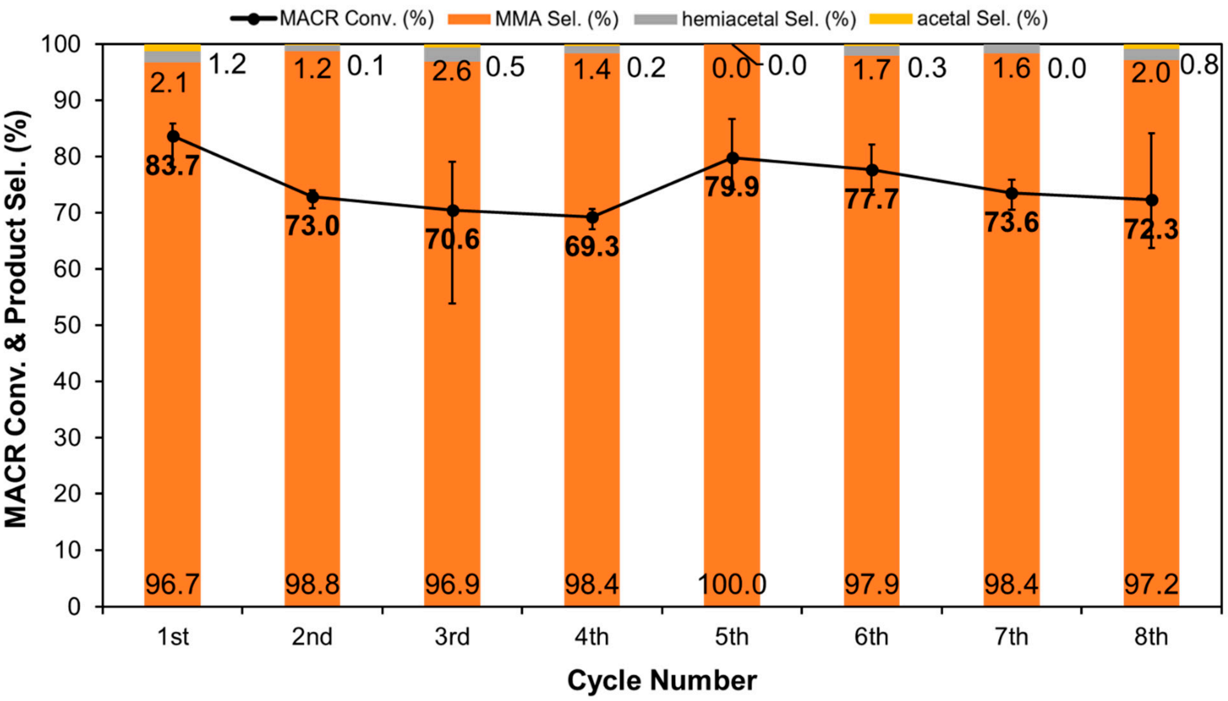

Figure S6. Recycle tests of 3AuM during the oxidative esterification of methacrylaldehyde with methanol for eight repetitive runs. Black dots indicate the conversion of methacrylaldehyde with error bars derived from three measurements. Orange bars with numbers at the bottom indicate the selectivity of methyl methacrylate. Each reaction is carried out at 80 °C for 1 h and follows reaction conditions similar to those in Entry 11 in Table 2.

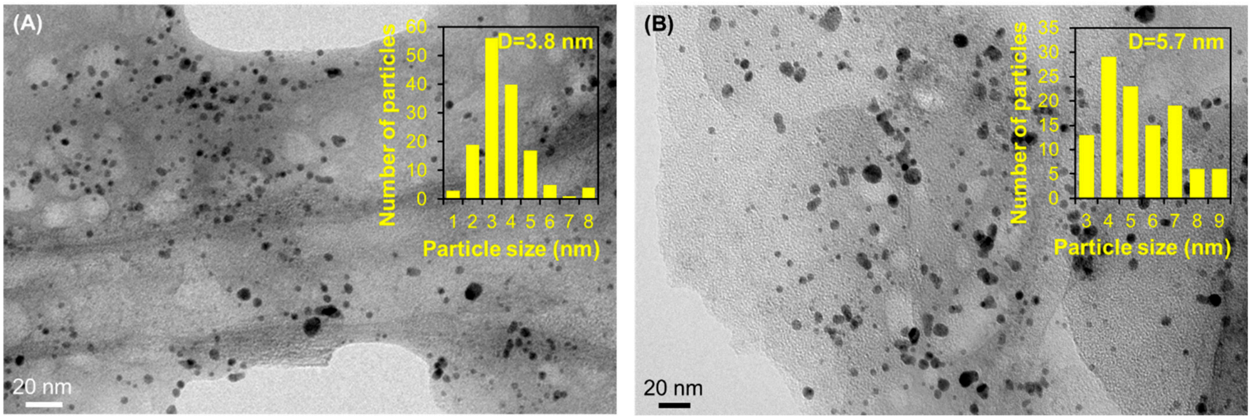

Figure S7. Transmission microscopy images of the 3AuM catalyst (**A**) before the reaction and (**B**) after eight repetitive reaction tests, as described in Figure S6. The average size of the Au nanoparticles in the fresh 3AuM catalyst before reaction is 3.8 nm (**A**, inset) and increases to 5.7 nm after eight repetitive reaction tests (**B**, inset). The particle size distribution graphs (insets) are obtained after counting 100 particles.

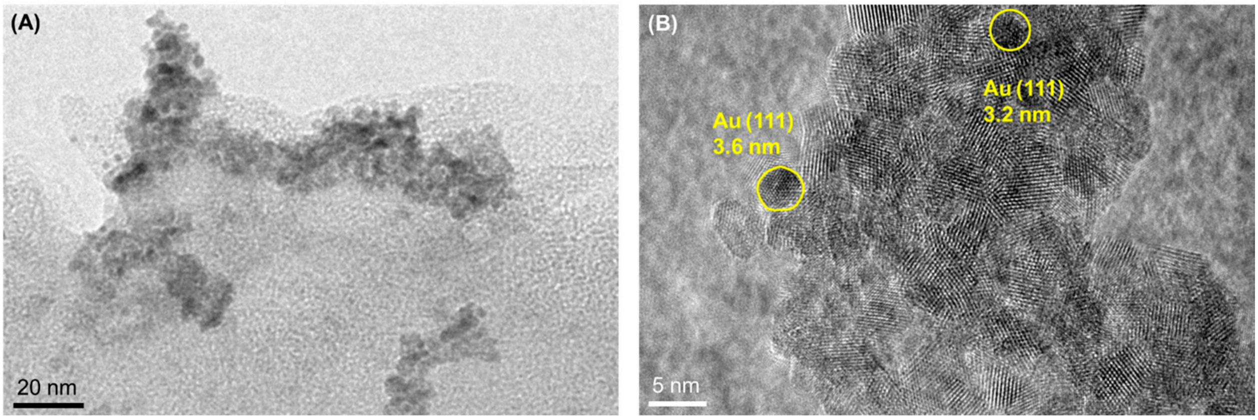

Figure S8. Transmission microscopy images of the 3AuCM catalyst at (**A**) low and (**B**) high magnifications after eight repetitive reaction tests, as described in Figure S5. No significant increase in the average size of Au nanoparticles is observed after eight repetitive reaction tests compared to that of the fresh catalyst shown in Figure. 2E–F.

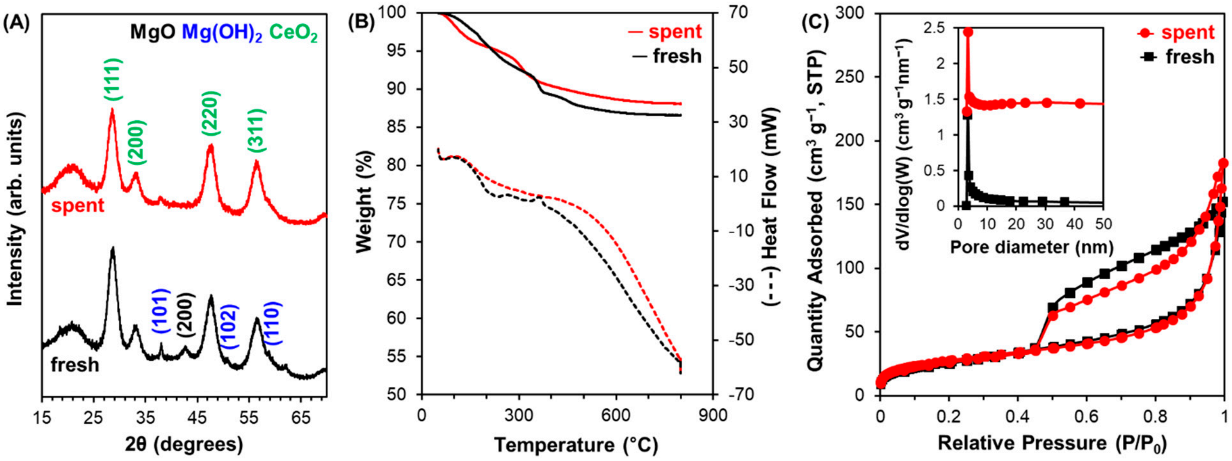

Figure S9. (**A**) X-ray diffraction patterns, (**B**) thermogravimetric analyses with differential scanning calorimetry, and (**C**) N_2 adsorption/desorption isotherms with pore size distributions (inset) of the 3AuCM catalyst before the reaction (black) and after eight repetitive reaction tests (red).
